# Assessment of congruence between co-occurrence and functional networks: A new framework for revealing community assembly rules

**DOI:** 10.1038/s41598-019-56515-7

**Published:** 2019-12-27

**Authors:** Gaëlle Legras, Nicolas Loiseau, Jean-Claude Gaertner, Jean-Christophe Poggiale, Dino Ienco, Nabila Mazouni, Bastien Mérigot

**Affiliations:** 1 Univ. Polynesie francaise, ifremer, ilm, ird, eio umr 241, tahiti, French Polynesia; 2 MARBEC, Univ Montpellier, CNRS, Ifremer, IRD, Sète, France; 3 University Grenoble Alpes, CNRS, Univ. Savoie Mont Blanc, LECA, Laboratoire d’Ecologie Alpine F-38000, Grenoble, France; 4 Institut de Recherche pour le Développement (IRD) – UMR 241 EIO (UPF, IRD, Ifremer, ILM) –Centre IRD de Tahiti, 98713 Papeete, French Polynesia; 5 Aix Marseille Université, CNRS/INSU, Université de Toulon, IRD, Mediterranean Institute of Oceanography (MIO) UM 110, 13288 Marseille, France; 6 IRSTEA Montpellier, UMR TETIS - F-34093, Montpellier, France

**Keywords:** Ecology, Community ecology, Ecological networks, Theoretical ecology

## Abstract

Describing how communities change over space and time is crucial to better understand and predict the functioning of ecosystems. We propose a new methodological framework, based on network theory and modularity concept, to determine which type of mechanisms (i.e. deterministic *versus* stochastic processes) has the strongest influence on structuring communities. This framework is based on the computation and comparison of two networks: the co-occurrence (based on species abundances) and the functional networks (based on the species traits values). In this way we can assess whether the species belonging to a given functional group also belong to the same co-occurrence group. We adapted the Dg index of Gauzens *et al*. (2015) to analyze congruence between both networks. This offers the opportunity to identify which assembly rule(s) play(s) the major role in structuring the community. We illustrate our framework with two datasets corresponding to different faunal groups and ecosystems, and characterized by different scales (spatial and temporal scales). By considering both species abundance and multiple functional traits, our framework improves significantly the ability to discriminate the main assembly rules structuring the communities. This point is critical not only to understand community structuring but also its response to global changes and other disturbances.

## Introduction

A fundamental question in ecology is whether and which assembly rules determine the structure of natural communities^1^. This knowledge is essential to understand processes and drivers structuring spatio-temporal distribution of communities (i.e. deterministic processes versus stochastic processes^2–4^. This understanding is particularly important in the current context of the ever-increasing pressure exerted by human activities at both local/regional (e.g. land-use modifications, pollution) and global scale (e.g. acidification of oceans, global warming) which imperils integrity of most ecosystems and capacity to deliver services to people^5^.

Nowadays, assembly rules of communities are mainly assessed in taking into account the functional diversity of organisms (i.e. the value and range of functional traits of the organisms in a given ecosystem^6^). Indeed, some studies have demonstrated that functional traits strongly contribute to determine species distribution in a complex environment^7–9^. Currently, three methodological approaches based on the use of functional diversity indices are mainly used in the literature for the common aim of assessing the relative influence of structuring (stochastic or deterministic) processes on communities: (i) FD index based on the construction of dendrograms from the distance matrix between species pairs^10–12^, (ii) FRic index based on the computation of a convex hull^13,14^, (iii) the n-dimensional hypervolume index^15,16^. However, while all these approaches have greatly improved our understanding of functioning of communities, they also suffer from strong limitations^17–20^. For example, FD index of Petchey and Gaston^10^ and convex hull volume of Cornwell *et al*.^13^ are only based on presence/absence data, while the structure and response of communities in the face of disturbance are strongly dependent of the distribution of species abundances.

Here, we have assumed that a new methodological approach based on the network theory might represent a fruitful alternative way. The use of network approaches to study complex communities has significantly advanced our understanding of ecological systems^21–23^. Currently, network approaches are used not only for studying food webs^24–26^ but also mutualistic and host-parasite interactions^4,27,28^. For instance, by considering species interactions network approaches have been used to highlight the importance of non-trophic edges between coexisting species^29^ or again to investigate the dynamic and the functional structure of trophic chains^25,30^. Otherwise, several studies demonstrated that comparing ecological networks along environmental gradients at multiple spatial scales may reveal species coexistence processes and community assembly rules (i.e. deterministic processes versus stochastic processes^2,3^). Surprisingly, while functional traits may strongly contribute to determine species distribution in a complex environment with several dimensions for space and time^7–9^, only rare studies coupled network approaches with the integration of functional information^9,24^, and even more rarely with several complementary functional traits (but see^31^). In addition, these approaches were mainly focused on the detection of significant associations (negative and positive) between species rather than on the detection of processes structuring the communities.

To overcome this gap, we introduce a new methodological approach based on the network theory and modularity concepts^32^. This approach allows to disentangle the main drivers (determinists, stochastics or both) of species co-occurrence (based on abundance or presence/absence data) taking into account multiple functional traits.

First, we provide a methodological explanation of our framework and highlight its accuracy for assessing community assembly rules. Second, we apply this framework on two data sets of bee’s and aquatic invertebrate’s communities representing different ecological contexts. We assess how our approach can detect assembly rules acting (1) at different spatial scales and (2) along temporal scale (i.e. before and after a disturbance event). We discuss the contribution of our approach for understanding of the mechanisms driving the community structure and to a larger extent, for understanding how communities will respond to global changes.

## Materials and Methods

### Methodological development

Our approach is based on the comparison of two networks: the functional network (based on the functional trait values of species) and the co-occurrence network (based on the number of co-occurrence between species). In the two networks the edges among groups do not represent interactions, such as species interactions used for most traditional networks in ecology, but the degree of functional complementary/redundancy (i.e. individuals sharing or not the same combination of values of functional traits), and species co-occurrence, for the functional and co-occurrence networks, respectively. Considering two distinct networks (i.e. functional and co-occurrence networks) and comparing then should allow to assess to which extent the species belonging to a given functional group also belong to the same co-occurrence group. When the two networks are similar, it means that deterministic processes such as environmental filtering are dominant^33^. Indeed, if functionally closed species tend to live in the same place, environmental conditions are expected to act as a major filter on species distribution^34,35^. In contrast, when the two networks are very different, it means that other deterministic processes occur, such as competition (i.e. limiting similarity process, following the theory of Mac Arthur & Levins^36^). Indeed, competitive exclusion will result in a pattern where species that are functionally similar are negatively associated^33,37,38^. Finally, if the distribution of species within both networks is not different from a random pattern of distribution, it means that either stochastic processes (i.e. neutral) are the main factors structuring the community^33,39,40^ or we have no clear dominance of one of the two deterministic processes (i.e. environmental filtering and competition).

**Figure 1:**
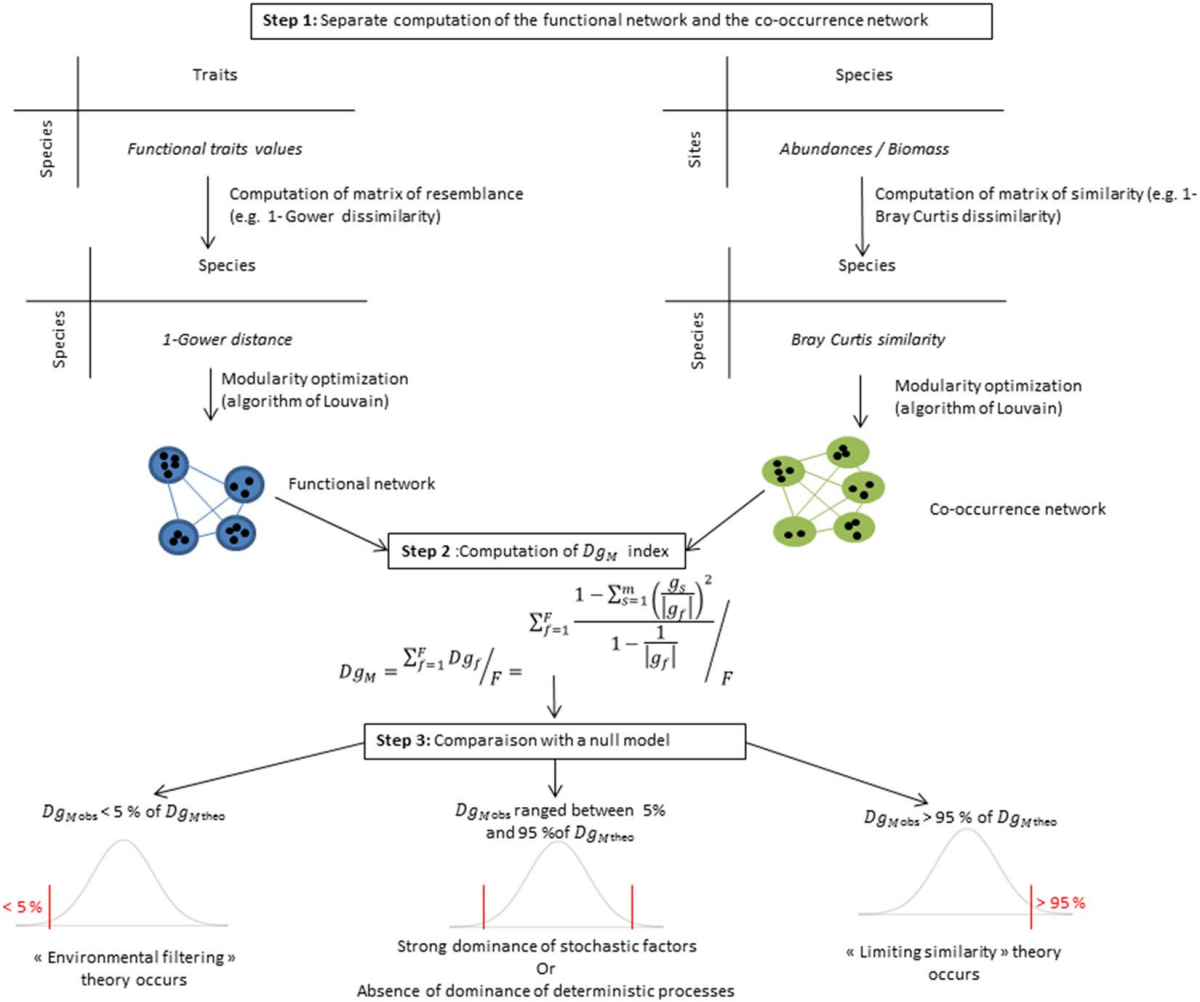
General framework to study the assembly rules of communities from functional and co-occurrences networks.

The methodology of our framework consists of three successive steps illustrated in Fig. 1 and detailed thereafter.

*Step 1*: Detection of the functional groups and the co-occurrence groups within each network

The first step of our approach consists in computing two different networks: the functional network and the co-occurrence network. Here, a network represents species as nodes, and the degree of species functional differences (i.e. if they share more or less the same functional trait values) or co-occurrence (i.e if they tend to co-exist) as edges. More precisely, the computation of these two different networks is done as follows:

- We defined the “functional network” by computing the functional trait resemblance matrix between species (computed from the species trait matrix, i.e. species in row, functional traits in column) with (1-standardized Euclidean distance) or (1-Gower index), according to the nature of variables, i.e. quantitative continuous or quantitative and qualitative mixed, respectively^17,41^. The values of this functional matrix are used to weight edges between species in the functional network where each species represent one node.
- We defined the “co-occurrence network” by computing the co-occurrence similarity matrix. This matrix is obtained by transposing the abundance matrix (i.e. the sites * species matrix) and then, by computing the similarity of Bray-Curtis between samples (i.e. 1- Bray-Curtis dissimilarity index, the number of times that these species have been seen together, weighted by their abundance^42^). The values of this similarity matrix are used to weight edges between species in the co-occurrence network. It is important to note that our method is not restricted to the use of Bray-Curtis index. Here we choose to use the similarity of Bray-Curtis for these desirable properties in ecology (e.g. combining the structural information on presence/absence with quantitative counts of species, non-consideration for double zero^43,44^) but other metrics could also be used according to the nature of available data and the objectives of each study (e.g. Jaccard, Canberra or Cao indices, etc.).

We first built the “functional network”. We computed the functional trait resemblance matrix among species, which embeds the degree of difference from a functional point of view among species pairwise. This matrix was computed based on the species trait matrix (i.e. species in rows, functional traits in columns) using the 1-standardized Euclidean distance or 1-the Gower index according to the nature of variables, i.e. quantitative continuous or quantitative and qualitative mixed, respectively^17,41^. The values of this matrix were then used to weight edges and their strength among species in the functional network where each species represents one node.

Secondly, we built the “co-occurrence network”. We computed the co-occurrence similarity matrix using the similarity Bray-Curtis on the species abundance matrix (i.e. species in column, sites in rows). 1- Bray-Curtis values represent the number of times that these species have been seen together, weighted by their abundance^42^. The values of this similarity matrix are used to weight edges and their strength among species in the co-occurrence network. Note that our method is not restricted to the use of the Bray-Curtis index. Here we choose to use the similarity of Bray-Curtis for these desirable properties in ecology (e.g. combining the structural information on presence/absence with quantitative counts of species, non-consideration for double zero^43,44^) but other metrics could also be used according to the nature of available data (e.g. species presence/absence data) and the objectives of each study (e.g. Jaccard, Canberra or Cao indices, etc.).

Finally, to define the respective groups of species for functional and co-occurrence networks, we use the concept of modularity in searching to optimize this modularity via the optimization algorithm of Louvain^45^. The modularity of a partition (Q) is a scalar value between −1 and 1 that measures the density of edges inside groups compared to edges between groups^32,46^. Thus, the higher value of modularity, the better the classification of species (or individuals) into groups is. We chose the algorithm of Louvain because it unveils hierarchies of groups and allows the discovery of sub-groups, sub-sub groups, etc. within groups^45^. This algorithm has been identified as one of three best algorithms for modularity detection (according to a comparative analysis that included 12 different algorithms^47^) and this method is currently used with success for several networks of different types, e.g. human brain network^48^, social network^49^, mobile phone networks^50^ and for large-sized networks (e.g. 4 M nodes and 100 M edges^50^, 21 M nodes^51^).

It is important to note that if no modular structure is found in the co-occurrence network, it means there is no significant structure in the dataset (i.e. in the co-occurrence matrix). Such a result assumes that no determinist processes prevails in the community (i.e. we have a dominance of stochastic processes or strong influence both environmental process and interspecies competition). Otherwise, if no modular structure is found in the functional network, it means that the choice of assessed functional traits is not accurate because it does not allow to differentiate the species/functional units between them.

*Step 2*: Assessing whether species affiliated to a given group of functional network also belong to the same group in the co-occurrence network.

The second step of this framework is to assess the congruence (i.e. the similarity) between the two networks (co-occurrence and functional networks, Fig. 1). This allows to assess whether the species affiliated to a defined functional group tend to be also in the same co-occurrence group. For this purpose, we measure an index of module diversity for each functional group *g*_*f*_ (derived from Gauzens *et al*.^25^):

$$D{g}_{f}=\frac{1-{\sum }_{s=1}^{m}{(\frac{{g}_{s}}{|{g}_{f}|})}^{2}}{1-\frac{1}{|{g}_{f}|}}$$

Where *g*_*s*_ is the number of functional entities (or species) of the functional group that belong to the co-occurrence group *S* and |*g*_*f*_| is the number of species in the functional group *g*_*f*_. We divide this index by ${1}-\frac{1}{|{g}_{f}|}$in order to range our index between 0 and 1. Then, we computed the *Dg*_*M*_ index which represents the mean of all *Dg*_*f*_ indices. *Dg*_*f*_ is 0 if all species of a functional group *g*_*f*_ belongs to the same co-occurrence group, and is 1 when all species of the functional group *g*_*f*_ belongs to different co-occurrence groups.

*Step 3:* Comparison of Dg_M_ index with null models

To investigate assembly rules driving the structure of communities, we compare the observed values of *Dg*_*M*_ to values distribution from a null model (Fig. 1). Null models allow comparing the deviation of empirical networks from random expectations and are expected to provide a more mechanistic understanding of the factors shaping ecological networks^52^. In null models, the partition into functional groups is identical to that obtained with our model (i.e. keeping the same number of co-occurrence groups and their respective sizes as the original dataset), but species are randomly distributed among co-occurrence groups. We then calculate a *p-value* from the iterations of null models (999 iterations in our study^40^). More specifically, we derive the *p-value* as the proportion of the null distribution of *Dg*_*M*_ index that is more extreme than the observed *Dg*_*M*_. If deterministic factors are lowly represented or if we have not a clear dominance of one of these factors, the observed *Dg*_*M*_ is expected to range between 5% and 95% of the null distribution^53^. In contrast, if niche-based processes (e.g. environmental filtering processes) prevail, the observed *Dg*_*M*_ should be significantly different from the null distribution. *Dg*_*M*_ index should be lower than expected at random (lower than 5% of the null distributions) if environmental filtering dominates. In contrast, if limiting similarity is the dominant process, we expect the *Dg*_*M*_ index to be higher than 95% of the null distribution^40^.

All computations implemented in this study were performed with R software (R Development Core Team, 2018) and in particular with ‘louvain’ function of the ‘modMax’ package. Randomizations were performed using the ‘sample’ R function. Code used to compute Dg_M_ index is available in Supplementary Material.

### Study cases

We illustrate the application of our approach on two datasets available in the literature. The datasets concern two different taxa (bee’s communities and aquatic invertebrate communities) and represent two different ecological situations frequently assessed (i.e. variation of communities’ structure along different spatial scales and after a disturbance event respectively).

#### Detection of assembly rules along different spatial scales

We used the data provided by Forrest *et al*.^54^ dealing with bee communities present in three types of habitats: four conventional farms (C), five organic farms (O) and seven natural areas (N) on the western slope of the Sacramento Valley, California. For each species, six functional traits were analyzed: two of them were continuous (intertegular distance and median day of year of flight season) and four of them were categorical (nest location, nesting behaviour, sociality and parasitic lecty). All these traits are known to affect the life-history of bees (see^54^ for more details regarding the sampling design). We applied our framework at regional scale (i.e. in considering all the types of habitats in the analysis) then at local scale (i.e. in considering each habitat separately) in order to assess which type of community-assembly factors dominates according to the spatial scale considered.

#### Temporal variation of assembly rules in response to a disturbance

We used published dataset of Bogan & Lytle^55^, also used in Boersma *et al*.^56^. An aquatic invertebrate community in a small and isolated stream (French Joe Canyon) in southeast Arizona was sampled before and after a severe drought and resultant stream drying event (8 years separating the two sampling periods). Seven categorical functional traits were selected that are associated with biological responses to drought in arid-land streams: body size, functional feeding group, dispersal ability, locomotion, voltinism, respiration and diapause^56–58^. We assessed the impact of this drying event on the community structure and composition in applying our framework on the invertebrate communities before and after the disturbance. The orders of magnitude of species abundance being strongly different (from few individuals to several thousand individuals), we applied a *log(n* + *1)* transformation on the abundance dataset.

### Sensitivity analyses

We first assessed the robustness of our framework to the number of traits by rerunning 1000 times all analyses using all combinations of two to N-1 traits for each dataset. We did not reduce the number of traits below two because we might have missed important dimensions of the functional space defining species niche. Secondly, we determined how number of species influences our framework in varying the number of species by steps of 10 species for invertebrate communities and by steps of 20 species for bee communities.

## Results

### Detection of assembly rules along different spatial scales

#### At regional scale (i.e. inter-habitats)

**Figure 2:**
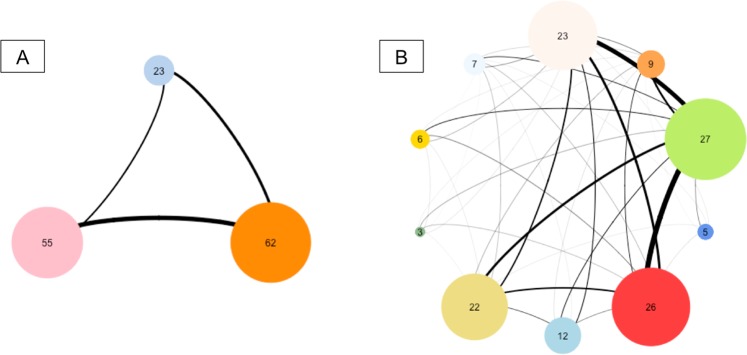
Schematic representation of networks obtained for bee communities after research of modularity with the algorithm of Louvain. The number inside the circle corresponds to the number of units composing each group and the width of edges is proportional to the strength of the similarity (i.e. the proximity) between the different groups. (**A**) Network obtained from the trait values resemblance matrix (referred as functional network). (**B**) Network obtained from the Bray-Curtis similarity matrix (referred as co-occurrence network).

We obtain a functional network composed of 3 groups (containing respectively 62, 55 and 23 species) and a co-occurrence network composed of 10 groups (algorithm of Louvain, Table 1, Fig. 2). The Dg_M_ index is 0.78. It is inferior to 5% of values obtain with null model (p-value = 0.04, Table 1), meaning that environmental filtering play a leading role in structuring bee communities at regional scale (i.e. inter-habitats).

**Table 1:** Assembly rules structuring Bee’s community according to the framework developed in this study.

Network	Spatial Scale	Habitat	Modularity	Number of groups	Value of Dg_M_ index	p-value
Functional network			0,09	3		
Co-occurrence network	Regional scale	Inter-habitat	0,3	10	0,78	**0,04**
	Local scale	Natural habitat	0,31	6	0,83	0,53
		Organic farms	0,29	6	0,81	0,44
		Conventional farms	0,22	5	0,76	0,16

Results are obtained for functional network and the different co-occurrence networks from Louvain algorithm (modularity optimization) along different spatial scales. The p-value represents the percentage of values of DgM from null model inferior to the DgM observed in each case. A percentage inferior to 5% highlights the presence of environmental filter acting on the ecological communities.

#### At local scale (i.e. intra-habitat)

For natural habitats, we obtain a co-occurrence network composed of 6 groups (containing 20, 24, 12, 22, 15 and 14 species, respectively). In combination with functional network (computed only from species present in natural habitat), the Dg_M_ index observed is 0.83 and not different from null model (Table 1). For both types of farms, the Dg_M_ index is respectively 0.81 for organic farms and 0.76 for conventional farms. As for natural habitats, these Dg_M_ values are not different from those obtained under null models (Table 1). These results mean that, at local scale, the dominance of one of deterministic processes is not highlighted.

### Temporal variation of assembly rules in response to a disturbance

#### Before the drying event

**Figure 3:**
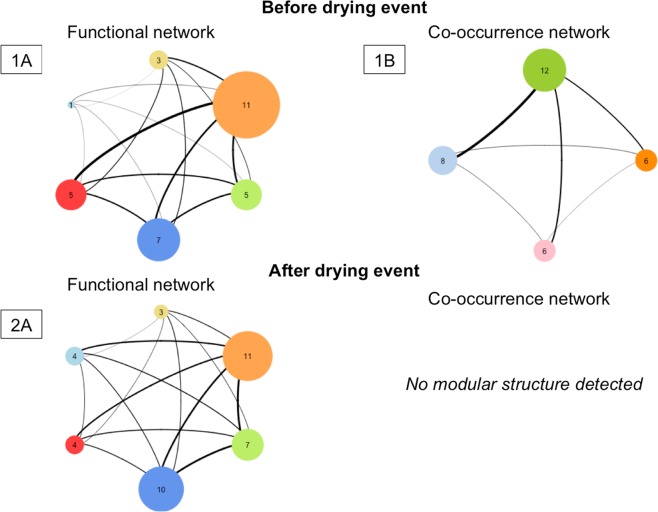
Schematic representation of networks obtained for aquatic invertebrate communities after research of modularity with the algorithm of Louvain. The number inside the circle corresponds to the number of units composing each group and the width of edges is proportional to the strength of the similarity (i.e. the proximity) between the different groups. (**A**) Network obtained from the trait values resemblance matrix (referred as functional network). (**B**) Network obtained from the Bray-Curtis similarity matrix (referred as co-occurrence network).

We obtained a functional network composed of 6 distinct groups (containing 11, 7, 5, 5, 3 and 1 species). For the co-occurrence network, it is composed of 4 groups (containing 12, 8, 6 and 6 species, see Fig. 3). The Dg_M_ index associated to these two networks (i.e. functional and co-occurrence networks) is 0.72 (see Table 2). It is inferior to 5% values obtained with null models (p-value = 0.04) supporting the hypothesis of a strong dominance of environmental filtering process.

**Table 2:** Assembly rules structuring aquatic invertebrate community along temporal scale according to the framework developed in this study.

	Modularity	Number of groups	Value of Dg_M_ index	p-value
*Before the drying event*				
**Functional network**	0,08	6	0,72	**0,04**
**Co-occurrence network**	0,14	4		
*After the drying event*				
**Functional network**	0.08	6		
**Co-occurrence network**	No modular structure			

Results are obtained for functional networks and the co-occurrence networks from Louvain algorithm (modularity optimization). The p-value represents the percentage of values of Dg_M_ from null model inferior to the Dg_M_ observed. A percentage inferior to 5% highlights the presence of environmental filter acting on the ecological communities.

#### After the drying event

We obtain a functional network composed of 6 groups (containing 11, 10, 7, 4, 4 and 3 species). In contrast, we found no modular structure for the co-occurrence network (see Table 2 and Fig. 3). This result assumes either the dominance of stochastic processes or the absence of a clear dominance of one of the two main deterministic processes (i.e. environmental filtering and competition).

### Sensitivity analysis

The sensitivity analyses related to the species number and the number of functional traits highlighted no relationship between these two parameters and the modularity of the network obtained, ie. no effect of species number and traits on the network modularity (all p-values are non-significant, see details in Supplementary Material). These results are valid for both datasets analyzed (i.e. bee communities and invertebrate communities). The variation observed for the network’s modularity for each level of each parameter (i.e. species richness or number of functional traits) is due to the fact that the communities slightly differed at each randomization.

## Discussion

Assessment of ecological networks can provide important insights into the relative importance of environmental filtering and coexistence mechanisms behind community assembly^4,59–63^. The framework developed here, based both on a co-occurrence and functional networks, allows to improve the assessment of processes of assembly occurring at different spatial and temporal scales. First, compared to the traditional approaches using functional diversity indices (e.g. convex hull volume of Cornwell *et al*.^13^, FD index of Petchey & Gaston^10^), the use of a co-occurrence network based on species abundance allows to take into account quantitatively the frequency of occurrence of different species that is particularly innovative.). This aspect worth emphasizing, because taking into account and quantifying the co-occurrences of species along the spatial and/or temporal dimensions is essential for understanding how the whole community is organized and functions^59^. Secondly, compared to approaches using networks to analyze community structure, our framework allows several complementary functional traits of species to be taken into account, which is very rare so far (but see^31^). However, our approach does not consider differences between individuals of the same group that can hide some ecological patterns (although these differences were taking into account during the step of network building). It can also be noted that a long time of computations can be observed when the dataset is large (e.g. important number of species) but this limitation only occurs during the computation of the algorithm of Louvain.

### Detection of assembly rules along different spatial scales

Through the assessment of bee communities in three types of habitats (i.e. natural habitat, organic farm and conventional farm), we highlight the fact that, at regional scale (i.e. between habitats), the environmental filtering process dominates (i.e. species with similar functional traits tends to co-occur in the same habitat). Conversely, at smaller scales (i.e. intra-habitat or local scale), we do not observe the predominance of determinist patterns. Thus, our results support the idea that the spatial scale has a great impact on the process dominating the community structure^4,64^. More precisely, these results support the theory according to which environmental filtering will constrain the pool of species co-occurring in a given region^65,66^. Sharing ecological traits, like adaptations to particular environmental conditions, is often a perquisite for two species to interact^9^. Thus, species turnover between habitats with different environmental conditions is recognized to be responsible for a large fraction of variation in community composition in space^67^. For this study case, we assume that the land-use modifications (due to the agricultural human activity) selected the bee species according to their trait values. Our results also support the results of other studies showing that, at local scales, interspecific competition is added to niche filtering and species tend to be more functionally distant^22^. By instance, Stubbs & Wilson^38^ have demonstrated the presence of limiting processes at local scale where some plants should differ in their traits values for the uses of water to persist and avoid strong competition between them. The lack of evidence concerning niche filtering process for both farms (i.e. organic and conventional farms) could be due to the degradation of natural habitat. Disturbances may modify assembly rules and blur assembly patterns. In disturbed habitats, the pressure applied by environmental conditions can be strong enough to allow the production of real competition patterns^68^. Unfortunately, in this case of the non-differentiation with the null model, it is not possible to know if it is due to stochastic processes (e.g. dispersal, natural disasters) or an overlap of determinist process. However, this point is not exclusive to our approach and concerns a great majority of methods based on the comparison of values with those a null model^33,69^. Developing an important methodological effort will be needed to resolve this drawback in the near future (but see^70^ for noticeable advances for presence-absence matrix).

### Temporal variation of assembly rules in response to a disturbance

We also demonstrate that our approach allows distinguishing patterns due to the impact of environmental disturbances on communities. In assessing the assembly rules structuring stream invertebrate communities before and after a severe drought in southeast Arizona for eight years^55^, we highlight differences in the structure of these communities. This difference could be explained by the fact that the drying event generates a more restricted access to resources for the species. Thus, the effects of competition between species are added to the effects of environmental conditions and modify the structure of communities. Indeed, changes of environmental conditions may strongly influence the identity and strength of species interactions by altering species’ spatial distribution^22^.

Overall, we believe that our framework should pave the way for a better understanding of the spatial and temporal structure of communities while considering co-occurrence, abundance and functional traits. Furthermore, our approach is applicable at multiple spatial scales that allow a more complete vision of patterns structuring the community following the different scales considered. Moreover, our results strongly support those of many studies arguing that the relative importance of determinist processes (i.e. environmental filtering and limiting similarity processes) structuring the communities vary according to the spatial scale considered^71^. Indeed, it is considered that the environmental filtering tends to predominate at larger spatial scale in constraining the establishment and the persistence of species according to their traits values in a given environment^34,72,73^. Conversely, the limiting similarity process is believed to occur at smaller spatial scale because this process translates the competition occurring between species and is added to the niche-filtering process^22^.

The approach proposed here, could be used for a wide range of situations, taxa and contexts, in both marine and terrestrial realms. It should contribute to several lively and emerging issues in community ecology. For instance, in considering each node as functional entities in the functional network (i.e. individuals sharing the same combination of values of functional traits) and not as species, it could be possible to integrate intraspecific functional information in our framework highlighting mechanism of co-existence between and within species. This is a critical point that enriches the potential of use of our approach in a context where a lot of studies have demonstrated that intraspecific traits variation (e.g. due to life stage, adaptation, etc.) can affect specific interactions such as competition, as well as overall ecological dynamics^9,74,75^.

Identification of rare functions and their roles in ecosystems is another emerging issue^76^. In assessing the number of species (or functional entities) and the composition of each functional module, our approach enables us to identify the species (or functional entities) which play rare functions in ecosystems. Moreover, analyses of positions of rare species and functions within co-occurrence and functional networks could provide insight on how rare functions shape structure of species co-occurrence^76,77^. It thus may help to predict the consequences of rare species extinction on ecosystem functioning^77–79^.

The choice of traits used is of primary importance for assessing functional diversity because it is dependent on both the ecological question addressed and the characteristics of the community studied^80^. It has thus to be done as a first step according to the knowledge and expertise of researchers. Then, it might be of ecological interest to assess the contribution of one particular trait on module patterns. An approach already used in functional diversity studies is to rerun analyses using all combinations of N-1 traits out of N^19^ which allow identification of relative trait contributions.

Finally, global change imposes modifications in the structure of species co-occurrence network for many ecosystems on Earth^4,81,82^. Our framework reconciling the approaches based on co-occurrence and those that consider functional traits is the first step towards the foundation of a unified framework. By improving our understanding of consequences of human-driven global changes on community assembly, network approaches can provide valuable insights in the assessment of processes and assembly rules structuring ecological communities. Network tools could also improve conservation sciences by bridging the gap between ecology and social sciences, allowing mix of different data in a unified framework.

## Supplementary information

Supplementary Information

## Data Availability

The datasets during the current study are available in *Forrest et al. 2015*, *Bogan and Lyttle 2011* and *Boersma et al. 2016*.
