# Supplementary material for: Assessment of congruence between co-occurrence and functional networks: A new framework for revealing community assembly rules
Source: Sci Rep. 2019 Dec 27;9:19996. doi: 10.1038/s41598-019-56515-7 (PMC6934466; doi:10.1038/s41598-019-56515-7)
Supplement: Supplementary file 1 — Supplementary Information [file 41598_2019_56515_MOESM1_ESM.docx]

Supplementary material for

**Assessment of congruence between co-occurrence and functional networks: a new framework for revealing community assembly rules**

Gaëlle Legras * ^a^, Nicolas Loiseau ^b,c^, Jean-Claude Gaertner ^d^, Jean-Christophe Poggiale ^e^, Dino Ienco ^f^, Nabila Mazouni ^a^ and Bastien Mérigot ^b^

^a^ Univ. Polynesie francaise, IFREMER, ILM, IRD, EIO UMR 241, Tahiti, Polynesie francaise

^b^ MARBEC, Univ Montpellier, CNRS, Ifremer, IRD, Sète, France

^c^ University Grenoble Alpes, CNRS, Univ. Savoie Mont Blanc, LECA, Laboratoire

d’Ecologie Alpine F-38000 Grenoble, France

^d^ Institut de Recherche pour le Développement (IRD) – UMR 241 EIO (UPF, IRD, Ifremer, ILM) –Centre IRD de Tahiti, 98713 Papeete French Polynesia

^e^ Aix Marseille Université, CNRS/INSU, Université de Toulon, IRD, Mediterranean Institute of Oceanography (MIO) UM 110, 13288, Marseille, France

^f^ IRSTEA Montpellier, UMR TETIS - F-34093 Montpellier, France

* Corresponding author: [legras.gaelle@gmail.com](mailto:legras.gaelle@gmail.com)

## SUPPORTING INFORMATION 1 : RCODE FOR COMPUTATION OF DgM INDEX

#' -------------------------------------------------------------------------- @Header

#'

#' @title COMPUTATION OF DgM INDEX

#'

#' @description

#' Assessment of congruence between co-occurrence and functional networks:

#' a new framework for revealing community assembly rules.

#' Legras G., Loiseau N., Gaertner J.C., Poggiale J.C., Ienco D., Mazouni N, Merigot B.

#' published in Nature: Scientific Reports

#'

#' @Script' author Gaelle LEGRAS, \email{legras.gaelle@gmail.com}

#'

#'

#' @date 2019/11/23

#'

#' @Rsession

#' R version 3.6.0 (2019-04-26) -- "Planting of a Tree"

#' Copyright (C) 2019 The R Foundation for Statistical Computing

#'

#' @attached base packages

#' [1] stats base

#' --------------------------------------------------------------------------

#' -------------------------------------------------------------------------- @Function

#'Input :dataframe with:

# col 1 : functional group

# col 2 : co-occurrence group

#output : values of Dgf pour for each functional group and last value is DgM

dgMcomputR<-function(trM){

trM<-as.data.frame(trM)

trM[,1]<-as.factor(trM[,1])

trM[,2]<-as.factor(trM[,2])

DgF<-NULL

Dg2<-NULL

for (i in 1:length(levels(trM[,1]))){

Sub<-subset(trM,trM[,1]==levels(trM[,1])[i])

for (j in 1:length(levels(trM[,2]))) Dg2[j]<-(sum(Sub[,2]==j)/dim(Sub)[1])^2

DgF[i]<-(1-sum(Dg2))/(1-1/dim(Sub)[1])

DgM<-mean(DgF)

names(DgM)<-"DgM"

}

return(c(DgF,DgM))

}

#' -------------------------------------------------------------------------- @ End of function

#' -------------------------------------------------------------------------- @Exemple

#'----Example with inverterbrate communities before disturbance

#'----Data avalaible from Bogan and Lytle (2011) (see References section)

library(ade4)

library(FD)

library(modMax)

library(ecodist)

#'---- Tr : species * traits matrix

#'---- Abund : species * log abundance site matrix before disturbance

#' ---- Functional network

disT<-as.matrix((1-gowdis(as.data.frame(Tr))))

gT<-louvain(as.matrix(disT))

names(gT[[3]])<-rownames(disT)

gT

save(gT,file="gT.RData")

#'---- Co-occurrence network

distab<-as.matrix(1-bcdist(Abund))

gab<-louvain(distab)

names(gab[[3]])<-rownames(distab)

gab

save(gab,file="gab.RData")

#'---- DgM index

com1<-as.data.frame(cbind(unlist(gab[[3]]),unlist(gT[[3]][names(gT[[3]])%in%names(gab[[3]])])))

colnames(com1)<-c("cooccurrence_grp","functional_grp")

com1

dg<-dgMcomputR(com1)

DgF<-dg[c(1:length(dgm1)-1)]

DgF

# '----- Dgf of each functional group

DgM<-dg[length(dg)]

DgM

#'----- For this example, DgM is 0.72.

#'--- Null models

dgnull<-NULL

for (i in 1:1000){

com_null<-com1

com_null[,2]<-sample(com1[,2],length(com1[,2]),replace=FALSE)

dgN<-dgMcomputR(com_null)

dgnull[i]<-dgN[length(dgN)]}

quantile(dgnull,probs=seq(0,1,0.05))

#'---- Determination of p-value from null models by comparaison of observed DgM with all DgMs obtained from null model.

#'---- if DgM observed is inferior to 5 percent of DgM values from null model : Presence of environmenalt filter (deterministic process)

#'---- if DgM observed is between 5 and 95 percent of DgM values from null model : Dominance of stochastic process and/or mix of both deterministic #'----processes (environmental filering and limiting similarity/competition)

#'---- if DgM observed is superior to 95 percent of DgM values from null model : Presence of limiting similarity/competition (deterministic process)

if (DgM<=quantile(dgnull,probs=seq(0,1,0.05))[[2]]) print ("environmental filter acting on communities")

if (DgM>=quantile(dgnull,probs=seq(0,1,0.05))[[20]]) print ("limiting similarity/competition acting on communities")

if (DgM>quantile(dgnull,probs=seq(0,1,0.05))[[2]]&DgM<quantile(dgnull,probs=seq(0,1,0.05))[[20]]) print ("Dominance of stochastic factors / Absence of dominance of deterministic processes")

#'---- Here p-value is 0.04. This result highlights the presence of environmental filter acting on invertebrate communities before disturbance.

Supporting Figure 1.

Sensitivity to the number of functional traits and the number of species on the computation of the network modularity for the dataset of bees communities (data of *Forrest et al. 2015*). Simulations were performed 1000 times for each level of the parameter “number of functional traits” or “species”. Functional traits (or species) were randomly selected at each simulation in the pool of functional traits available (or pool of total species of bees communities, respectively). We found no significant statistical relations between these parameters and the network modularity (analysis of variance ANOVA, d.f.= 3726, p-value =0.89 and d.f.=6993, p-value = 0.56 for number of functional traits and number of species respectively).

Supporting Figure 2.

Sensitivity to the number of species on the computation of the network modularity for the dataset of aquatic invertebrate communities (data of *Bogan and Lytle, 2011*). Species were randomly selected at each simulation in the pool of total species of invertebrate communities. Simulations were performed 1000 times for each number of species tested. We found no significant statistical relations between the number of species and the network modularity (analysis of variance ANOVA, d.f.= 2997, p-value = 0.81 and , d.f.=2997 , p-value=0.69 for before disturbance and after disturbance respectively).

Supporting Figure 3.

Sensitivity to the number of functional traits on the computation of the network modularity for the dataset of aquatic invertebrate communities (data of *Bogan and Lytle, 2011*). Functional traits were randomly selected at each simulation in the pool of functional traits available for the invertebrate communities. Simulations were performed 1000 times for each number of functional trait tested. We found no significant statistical relations between the number of functional traits and the network modularity (analysis of variance ANOVA, d.f.=5997 , p-value= 0.69).
